# Supplementary material for: LDLR c.89_92dup: a novel frameshift variation in familial hypercholesterolemia
Source: Lipids Health Dis. 2024 Jun 12;23:182. doi: 10.1186/s12944-024-02173-2 (PMC11167941; doi:10.1186/s12944-024-02173-2)
Supplement: Supplementary file 2 — Supplementary Material 2 [file 12944_2024_2173_MOESM2_ESM.pdf]

iThenticate

manuscript_V3_查重
May 31, 2024
2703 words / 14559 characters

# manuscript_V3_查重

## Sources Overview

**13%**
OVERALL SIMILARITY

| | | |
|---|---|---|
| 1 | www.freshpatents.com<br>INTERNET | 1% |
| 2 | www2.mdpi.com<br>INTERNET | 1% |
| 3 | Tadao Iwasaki, Sadao Takahashi, Mitsuaki Ishihara, Masafumi Takahashi et al. "The important role for βVLDLs binding at the fourth cyst...<br>CROSSREF | 1% |
| 4 | www.mdpi.com<br>INTERNET | <1% |
| 5 | assets.researchsquare.com<br>INTERNET | <1% |
| 6 | jamanetwork.com<br>INTERNET | <1% |
| 7 | Fangyuan Li, Pucong Ye, Yu Hao, Juan Du, Hang Zhang, Zengtao Wang, Xumin Wang, Hui Zeng, Yaluan Ma, Jie Lin. "A PCSK9 inhibitor i...<br>CROSSREF | <1% |
| 8 | Prakash Kumar, Shashi Ranjan Prasad, Anushil Anand, Rajneesh Kumar, Sajalendu Ghosh. "Prevalence of familial hypercholesterolemi...<br>CROSSREF | <1% |
| 9 | translational-medicine.biomedcentral.com<br>INTERNET | <1% |
| 10 | www.hindawi.com<br>INTERNET | <1% |
| 11 | I. Ramasamy. "Update on the molecular biology of dyslipidemias", Clinica Chimica Acta, 2016<br>CROSSREF | <1% |
| 12 | worldwidescience.org<br>INTERNET | <1% |
| 13 | discovery.ucl.ac.uk<br>INTERNET | <1% |
| 14 | www.science.gov<br>INTERNET | <1% |
| 15 | lipidworld.biomedcentral.com<br>INTERNET | <1% |
| 16 | www.researchgate.net<br>INTERNET | <1% |
| 17 | Jeenduang, N.. "Molecular modeling of D151Y and M391T mutations in the LDL receptor", Biochemical and Biophysical Research Com...<br>CROSSREF | <1% |

18 Zhyldyz T Kabaeva. "Systematic analysis of the regulatory and essential myosin light chain genes: genetic variants and mutations in h... <1%
CROSSREF

19 docksci.com <1%
INTERNET

20 oxfordjournals.org <1%
INTERNET

21 uu.diva-portal.org <1%
INTERNET

22 www.frontiersin.org <1%
INTERNET

23 www.spandidos-publications.com <1%
INTERNET

24 Thais Kristini Almendros Barbosa, Rosario Dominguez Crespo Hirata, Glaucio Monteiro Ferreira, Jéssica Bassani Borges et al. "LDLR m... <1%
CROSSREF

25 Marianne Abifadel, Catherine Boileau. "Genetic and molecular architecture of familial hypercholesterolemia", Journal of Internal Medici... <1%
CROSSREF

**Excluded search repositories:**

- None

**Excluded from document:**

- Bibliography

**Excluded sources:**

- None

**Excluded preprints**

- None

## Abstract

**Background:** Familial hypercholesterolemia (FH) is a common inherited metabolic disease that causes premature atherosclerosis, cardiovascular disease, and even death at a young age. Approximately 95% of FH-causing genetic variants that have been identified are in the *LDLR* gene. However, only 10% of the FH population worldwide has been diagnosed and adequately treated, due to the existence of numerous unidentified variants, uncertainties in the pathogenicity scoring of many variants, and a substantial number of individuals lacking access to genetic testing.

**Objective:** The aim of this study was to identify a novel variant in the *LDLR* gene that causes FH in a Chinese family, thereby expanding the spectrum of FH-causing variants.

**Methods:** Patients were recruited from Beijing Anzhen Hospital, Capital Medical University. FH diagnosis was made according to the Dutch Lipid Clinical Network (DLCN) criteria. Whole-exome sequencing (WES) was conducted to identify the FH-causing variant in the proband, and amplicon sequencing was used to verify the variant in his family members.

**Results:** A three-generation Chinese family was recruited, and two FH patients were clinically diagnosed, both without known FH-causing variants. These two FH patients and another possible patient carried a novel variant, NC_000019.9(NM_000527.5):c.89_92dup (NP_000518.1:p.Phe32Argfs*21), in the ligand-binding domain of the low-density lipoprotein (LDL) receptor that led to a frameshift. The FH adults in the family showed severe clinical symptoms and statin therapy resistance.

**Conclusion:** This study identified a novel pathogenic *LDLR* variant, c.89_92dup, associated with severe FH clinical manifestations and statin therapy resistance.

**Keywords:** familial hypercholesterolemia; low-density lipoprotein receptor; c.89_92dup variant; ligand-binding domain; whole-exome sequencing

## Background

Familial hypercholesterolemia (FH) is an autosomal dominant metabolic disorder that is characterized by high serum levels of low-density lipoprotein (LDL) cholesterol [1]. The prevalence of FH is approximately 1 in 250~500 worldwide [2, 3], which is higher than that of most other inherited diseases. In patients carrying heterozygous variants, serum LDL cholesterol levels are typically above 5.0 mmol/L, which is 2 times greater than those in unaffected individuals within the same family [4]. In patients carrying homozygous variants, serum LDL cholesterol levels frequently exceed 13.0 mmol/L [5]. Long-term hypercholesterolemia can manifest as tendon xanthomas and arcus cornealis [1]. When left untreated, FH patients often suffer from premature atherosclerosis, cardiovascular disease (CVD), and even death at a very young age [6, 7]. In China, individuals with FH have a 15-fold increased risk of developing CVD compared to those without FH [8].

Known FH-causing variants are found in several genes that are associated with the removal of LDL particles from the circulation by hepatocytes, such as *LDL receptor* (*LDLR*, >95%), *apolipoprotein B* (*APOB*, 2–11%), and *proprotein convertase subtilisin/kexin type 9* (*PCSK9*, <1%) [1, 9]. The *LDLR* gene is located at 19p13.2 and comprises 18 exons spanning 45 kilobases (kb). It encodes an 860 amino acid protein that consists of five functional domains, including a ligand-binding domain, an epidermal growth factor (EGF) precursor homology domain, an O-linked polysaccharide domain, a transmembrane domain, and an intracellular domain [10]. According to the statistical data from the ClinVar database [11] (http://www.ncbi.nlm.nih.gov/clinvar/), more than 40% of FH-causing variants

are located in the EGF precursor homology domain, which impairs lipoprotein release and receptor recycling [12]. Another 40% of variants are located in the ligand-binding domain and therefore influence the binding of lipoproteins to LDL and VLDL (very low-density lipoprotein) [13]. No more than 20% of variants are scattered across the other three domains and introns [14].

According to the Familial Hypercholesterolemia Foundation and the World Heart Federation, only 10% of the FH population has been diagnosed and adequately treated [15]. Diagnoses are missed partially due to the existence of numerous unidentified variants, uncertainties in the pathogenicity scoring of many variants, and a substantial number of individuals lacking access to genetic testing. In this study, a three-generation Chinese FH family was diagnosed using the Dutch Lipid Clinical Network (DLCN) criteria [16]. A novel frameshift variant, c.89_92dup, was identified in the ligand-binding domain of the LDLR protein via whole-exome sequencing (WES). This variant broadens the spectrum of FH-causing variants, facilitating the diagnosis of individuals with FH.

# Materials and Methods

## Study Subjects

The subjects were recruited from an FH cohort at Beijing Anzhen Hospital, Capital Medical University. The proband was hospitalized and clinically diagnosed with FH according to the DLCN diagnostic criteria. Then, cascade screening was conducted for his family members. Six participants were included in the study, and all the subjects underwent a detailed physical examination. This study has been granted approval by the Ethics Committee of Beijing Anzhen Hospital (2022038X). All participants willingly took part in the research and provided their signatures on informed consent forms.

**Isolation of peripheral blood mononuclear cells (PBMCs)**

Peripheral blood was collected from the proband and his family members into Vacutainer tubes containing EDTA-2K and processed for PBMC isolation in a short time. Following plasma extraction by centrifugation (2000 rpm, 10 min), the precipitate was resuspended in an equal volume of phosphate-buffered saline (PBS). The sample was then layered onto Ficoll-Paque (GE Healthcare, Marlborough, MA, USA) and subsequently processed in accordance with the manufacturer's instructions. DNA was extracted from approximately 1x$10^6$ PBMCs for genetic analysis.

**Biochemical analysis**

A Roche COBAS 701 analyzer was used to assess the serum total cholesterol (TC), total triglyceride (TG), high-density lipoprotein (HDL) cholesterol, non-HDL cholesterol, low-density lipoprotein (LDL) cholesterol, small dense LDL cholesterol, lipoprotein (a), apolipoprotein A1, and apolipoprotein B levels. The LDL cholesterol level was measured using a direct assay (LDL-Cholesterol Gen.3 (LDLC3), Roche Diagnostics).

**WES library preparation and sequencing**

A Qiagen DNeasy kit (Qiagen, USA) was used to extract genomic DNA from the peripheral blood cells of the proband. After sonication, the DNA fragments were subjected to end repair and were supplemented with adapters at both ends. Following library amplification, the fragments were captured and enriched using an exon array. After washing to remove the nonenriched fragments, the remaining DNA was amplified and subjected to whole-exome sequencing using the Illumina HiSeq 3000 platform in paired-end sequencing mode (analysis performed by the Nantong KingMed Center for Clinical Laboratory).

The raw reads were mapped to the reference human genome (hg19) using Bowtie2 [17]. After filtering low-quality nucleotides (quality < 30) and reads with lengths less than 18 bp by Trimmomatic [18], the qualified reads were further processed with SAMtools to call SNPs using mpileup [19]. SNPs detected on all known genes were annotated with ANNOVAR software [20].

### Amplicon sequencing

Primers I and II were designed according to the *LDLR* sequence in the GenBank nucleotide sequence database (https://www.ncbi.nlm.nih.gov/nucleotide/) and had the following sequences: Primer I, 5′-TTTATTAATGTGCATGGAAGTTCT-3′; and Primer II, 5′- AGACCAGAAATTCAAGACCAGC-3′. With polymerase chain reaction (PCR) technology, amplicons containing the variant were amplified from equal amounts of DNA samples. Sanger sequencing was employed to validate the identified variant in other members of the family. Amino acid conservation near the variant position was examined using MEGA 11.0 software.

### Open reading frame prediction of the *LDLR* variant

The NCBI ORF Finder (https://www.ncbi.nlm.nih.gov/orffinder) was used to identify changes in the open reading frame (ORF) for the NM_000527.5 sequence with the c.89_92dup variant.

## Results

### Clinical characteristics of the FH family

The proband was a 32-year-old man who was hospitalized for "unstable angina" and clinically diagnosed with FH according to the DLCN criteria (Table 1). The patient showed severe clinical symptoms of FH, including arcus cornealis and tendon xanthoma (Figure 1). Coronary angiography revealed triple-vessel disease involving the right coronary artery (RCA), left circumflex artery (LCX),

and left anterior descending artery (LAD), with complete occlusion in the distal segment of the circumflex artery. The patient was insensitive to statin therapy. Before treatment, his LDL cholesterol level reached 8.12 mmol/L. After two years of treatment with 20 mg atorvastatin plus 10 mg ezetimibe, his LDL cholesterol level remained high at 6.92 mmol/L. Furthermore, he suffered an acute myocardial infarction while on the lipid-lowering regimen. Fortunately, the patient responded well to a PCSK9 inhibitor. After one month of treatment with evinacumab (140 mg every 2 weeks), his LDL cholesterol level decreased to 0.85 mmol/L.

Upon investigation of the proband's family history, it was discovered that his father (I.1) also met the clinical criteria for FH (Table 1), with an untreated LDL cholesterol level of 6.75 mmol/L. In his forties and again in his fifties, the father underwent arterial stent implantation for acute coronary syndrome (ACS). The elder daughter (III.1) of the proband was seven years old and exhibited significantly higher levels of LDL cholesterol (4.29 mmol/L). Neither the proband's mother (I.2) nor the younger daughter (III.2) exhibited FH-related clinical symptoms. Table 2 shows the family's lipid profile and clinical manifestations.

**Variant identification in the FH family**

In the proband (II.1), a novel variant, c.89_92dup, was identified in exon 2 of the *LDLR* gene by WES (Figure 2A). No variants were identified in other FH-causing genes or other exons of the *LDLR* gene. This duplication variant was located in a phylogenetically conserved region of the ligand-binding domain (Figure 2B) and produced a frameshift in the coding sequence. The frameshift ORF included an arginine amino acid variant at the 32nd position, which replaced the wild-type phenylalanine, and translation terminated at the 52nd amino acid (Figure 2C). By predicting potential ORFs longer than 50

codons in the *LDLR* variant, four other ORFs were discovered to include novel translation start codons (TSCs, Figure 2C). The longest predicted protein was truncated at the 263rd amino acid and lacked the first five repeats and a portion of the sixth repeat of the ligand-binding domain of LDLR.

## Variant Segregation Analysis

Amplicon sequencing confirmed that the c.89_92dup variant was carried by the proband's father (I.1) and elder daughter (III.1), while the proband's mother (I.2) and younger daughter (III.2) had the wild-type *LDLR* gene. Figure 3 displays the pedigree of this FH family, showing that dyslipidaemia was correlated with the segregation arrangement of the frameshift *LDLR* variant (Table 1). Therefore, the c.89_92dup variant was determined to be the pathogenic variant for this FH family.

# Discussion

This study reported a family in which the proband and his father were clinically diagnosed with FH according to the DLCN criteria, and the proband's elder daughter exhibited significantly higher levels of LDL cholesterol than her contemporaries. WES and amplicon sequencing confirmed that these three patients were found to carry the same variant, c.89_92dup, which was consistent with autosomal dominant inheritance. This duplication variant was situated within the ligand-binding domain of LDLR, which consists of seven repeats of 40 residues each and contains the binding sites for apoproteins B-100 (apoB-100) and E (apoE) [21, 22]. These apoproteins are essential components of LDL and VLDL particles. Since this frameshift variant is absent from control populations (Genome Aggregation Database, 1000 Genomes Project, and Human Gene Mutation Database) [23-25] and

segregated with a phenotype in 3 informative meiosis in the proband's family, the c.89_92dup variant was classified as pathogenic (PVS1, PM2, PP1 and PP4) according to the ACMG guidelines [26, 27].

Five ORFs in the *LDLR* gene with the c.89_92dup variant were found to be longer than 50 amino acids. Among them, ORF1 had the same TSC as the wild-type *LDLR* transcript. This ORF potentially encoded a truncated protein consisting of only 32 amino acids at the N-terminus of LDLR, along with 20 novel amino acids from the frameshift. Since dozens of RNA splice sites are located downstream of the premature termination codons (PTCs) in ORF1, the unremoved exon–exon junction complexes (EJCs) binding to mRNA should trigger nonsense-mediated mRNA decay (NMD) to degrade the transcript. ORF2 was predicted to produce an N-terminal truncated protein, adopting an ATG codon at positions 794-796 of the wild-type *LDLR* coding sequence (CDS) as the TSC. This truncated protein lacked the first five repeats and a portion of the sixth repeat of the ligand-binding domain of LDLR. The fifth repeat is required to bind β-VLDL, and the second, third, and sixth repeats are required for maximal binding of LDL [13]; therefore, the encoded protein produced by ORF2 lacks most of its ligand-binding functions. Additionally, the amino acid sequences encoded by the other three potential ORFs (ORF3-5) do not resemble those of the wild-type LDLR protein. In summary, although the impact of the c.89_92dup variant on LDLR protein sequence and abundance still needs to be verified, this variant may severely impair LDLR function in patients.

Consistent with the ORF analyses, patients with the c.89_92dup variant had more severe clinical symptoms. In the reported FH family, the proband and his father had LDL cholesterol levels of 8.12 mmol/L and 6.75 mmol/L, respectively. These findings were significantly greater than the median level of 5.43 mmol/L (IQR: 4.32-6.72 mmol/L) observed in adults with heterozygous FH who did not use lipid-lowering medications [4]. In addition, the proband suffered an acute myocardial infarction at the

early age of 32, while his father had a myocardial infarction in his forties and a recurrent episode of ACS at the age of 55. Both patients met the diagnostic criteria for severe FH [28].

More importantly, patients with the c.89_92dup variant seemed to be insensitive to statin therapy. Here, the proband's LDL cholesterol level decreased by only 15% after two years of treatment with 20 mg atorvastatin plus 10 mg ezetimibe. This observation further showed that the duplication variant significantly impaired LDLR function, resulting in an ineffective reduction in circulating cholesterol levels despite inhibiting cholesterol synthesis and absorption. However, the proband responded well to a PCSK9 inhibitor. When taken with statins and ezetimibe, coadministration of a PCSK9 inhibitor reduced the patient's LDL cholesterol level by up to 90%. Because a heterozygous FH individual has the homologous wild-type *LDLR* gene, a PCSK9 inhibitor increases LDLR abundance on the surface of hepatocytes, thus enhancing its ability to clear LDL cholesterol [29, 30]. Therefore, PCSK9 inhibitors are recommended for lipid-lowering therapy in patients carrying the *LDLR* c.89_92dup variant at an early stage.

## Strengths and limitations of the study

Using WES and variant segregation analysis, a frameshift variant, c.89_92dup, was identified as a novel pathogenic variant for severe FH with a poor response to statin therapy. This finding contributes to the clinical diagnosis and treatment of FH patients. Further molecular testing of the residual activity of this LDLR variant could provide valuable insights into its biological functions.

## Conclusions

The current study identified a novel pathogenic *LDLR* variant, c.89_92dup, associated with severe FH

clinical manifestations and statin therapy resistance. This study expands the spectrum of FH-causing variants and provides assistance for disease screening and individualized treatment. For optimal management, FH patients with the c.89_92dup variant should receive PCSK9 inhibitors in a timely manner.
